# Environmental Nephrotoxicity Across the Life Course: Oxidative Stress Mechanisms and Opportunities for Early Intervention

**DOI:** 10.3390/antiox14101205

**Published:** 2025-10-04

**Authors:** Chien-Ning Hsu, Chih-Yao Hou, Yu-Wei Chen, Guo-Ping Chang-Chien, Shu-Fen Lin, You-Lin Tain

**Affiliations:** 1 Department of Pharmacy, Kaohsiung Chang Gung Memorial Hospital, Kaohsiung 833, Taiwan; cnhsu@cgmh.org.tw; 2 Department of Pharmacy, Kaohsiung Municipal Ta-Tung Hospital, Kaohsiung 801, Taiwan; 3 School of Pharmacy, Kaohsiung Medical University, Kaohsiung 807, Taiwan; 4 Department of Seafood Science, National Kaohsiung University of Science and Technology, Kaohsiung 811, Taiwan; chihyaohou@nkust.edu.tw; 5 Department of Food Science and Biotechnology, National Chung Hsing University, Taichung 402, Taiwan; d112043001@mail.nchu.edu.tw; 6 Department of Pediatrics, Kaohsiung Chang Gung Memorial Hospital, Kaohsiung 833, Taiwan; 7 Center for Environmental Toxin and Emerging-Contaminant Research, Cheng Shiu University, Kaohsiung 833, Taiwan; guoping@csu.edu.tw (G.-P.C.-C.); 6101@gcloud.csu.edu.tw (S.-F.L.); 8 Super Micro Mass Research and Technology Center, Cheng Shiu University, Kaohsiung 833, Taiwan; 9 Institute of Environmental Toxin and Emerging-Contaminant, Cheng Shiu University, Kaohsiung 833, Taiwan; 10 Department of Pediatrics, Kaohsiung Municipal Ta-Tung Hospital, Kaohsiung 801, Taiwan; 11 College of Medicine, Chang Gung University, Taoyuan 333, Taiwan

**Keywords:** oxidative stress, antioxidants, hypertension, chronic kidney disease, pollutants, endocrine-disrupting chemicals, developmental origins of health and disease

## Abstract

Chronic kidney disease (CKD) affects nearly 10% of the global population, ranks among the top ten causes of death, and often progresses silently to end-stage disease without timely intervention. Increasing evidence indicates that many adult-onset cases originate in early life through adverse influences on kidney development, a process termed kidney programming within the Developmental Origins of Health and Disease (DOHaD) framework. Environmental pollutants are now recognized as key drivers of kidney injury across the life course. Heavy metals, air pollutants, plastic contaminants such as bisphenol A, phthalates, and micro/nanoplastics—as well as biocontaminants like mycotoxins and aristolochic acid—and chronic light pollution can accumulate in kidney tissue or act systemically to impair function. These exposures promote oxidative stress, inflammation, and endothelial and circadian disruption, culminating in tubular injury, glomerular damage, and fibrosis. Notably, early-life exposures can induce epigenetic modifications that program lifelong susceptibility to CKD and related complications. Oxidative stress is central to these effects, mediating DNA, lipid, and protein damage while influencing developmental reprogramming during gestation. Preclinical studies demonstrate that antioxidant-based interventions may mitigate these processes, providing both renoprotective and reprogramming benefits. This review explores the mechanistic links between environmental pollutants, oxidative stress, and kidney disease and highlights antioxidant strategies as promising avenues for prevention and intervention in vulnerable populations.

## 1. Introduction

Chronic kidney disease (CKD) poses a major global health burden, affecting approximately 10% of the world’s population and ranking among the top 10 leading causes of death worldwide [1,2]. Notably, CKD is largely preventable, and its progression to end-stage disease can be delayed through timely and effective interventions—particularly when initiated early [3]. In light of this, the global kidney health community has increasingly emphasized the importance of early identification of individuals at high risk—including children, pregnant women, and their fetuses—and the implementation of targeted prevention strategies across the life course [3,4]. Mounting evidence suggests that adult-onset CKD often originates in early life due to adverse influences on kidney development, a process known as kidney programming [5,6]. This concept is rooted in the broader framework of the Developmental Origins of Health and Disease (DOHaD) [7,8].

Common toxicants [9,10]—including heavy metals (e.g., lead, cadmium) [11], air pollutants (e.g., PM2.5) [12], emerging plastic contaminants such as bisphenol A (BPA), phthalates, and microplastics/nanoplastics (MPs/NPs) [13,14,15], as well as environmental stressors like chronic light pollution—can bioaccumulate in kidney tissues or exert systemic effects that impair kidney function. These exposures induce oxidative stress, trigger chronic inflammation, disrupt endothelial and circadian-regulated pathways, and contribute to tubular injury, glomerular damage, and interstitial fibrosis [9,10,11,12,13,14,15].

Early-life exposure to environmental pollutants is particularly concerning, as it can induce epigenetic alterations and developmental programming that elevate the long-term risk of CKD and related complications such as hypertension [10,16]. Moreover, pollutant exposure may exacerbate oxidative stress-mediated tissue injury. Epidemiological studies have consistently linked such exposures to reduced glomerular filtration rate (GFR), proteinuria, and accelerated CKD progression. Thus, environmental pollutants constitute a modifiable risk factor with important implications for both clinical management and public health policy.

Oxidative stress is a central pathogenic mechanism in pollutant-induced kidney disease [17,18,19]. Many environmental toxicants stimulate excessive production of reactive oxygen and nitrogen species (ROS/RNS), disrupting redox homeostasis and causing oxidative damage to DNA, proteins, and lipids in renal tissues. During pregnancy, elevated oxidative stress may also trigger epigenetic modifications that alter gene expression and contribute to the developmental programming of CKD [20]. Conversely, antioxidant therapies have demonstrated both renoprotective and reprogramming effects in preclinical models of kidney programming linked to early-life environmental exposures [21]. CKD is influenced not only by environmental toxicants but also by occupational exposures—metals, solvents, heat, and work-related stress—that cause renal injury through oxidative stress and mitochondrial dysfunction [22]. Targeting these oxidative stress pathways provides a promising strategy for early detection, prevention, and intervention, particularly in vulnerable populations such as children, pregnant women, and individuals with pre-existing kidney disease.

This narrative review summarizes current evidence on the role of environmental pollution in kidney disease arising from both well-known adult risk factors and early-life developmental exposures, with a particular focus on oxidative stress mechanisms. It also highlights the potential therapeutic benefits of antioxidant-based strategies for the treatment and prevention of pollution-related kidney injury.

## 2. Materials and Methods

Considering the breadth of disciplines and variability in the existing studies, a narrative review was selected over a systematic or scoping approach to allow for an integrative exploration of emerging concepts spanning nephrology, developmental biology, redox signaling, toxicology, and environmental health.

This review aims to collect current evidence on the role of oxidative stress in mediating the effects of environmental pollutants on kidney disease risk, with a particular focus on developmental programming and redox-targeted interventions. A comprehensive literature search was conducted using PubMed, Scopus, and Web of Science through June 2025. Search terms included combinations of: “oxidative stress,” “reactive oxygen species,” “reactive nitrogen species,” “nitric oxide,” “pollutants,” “endocrine-disrupting chemicals,” “organophosphate flame retardants,” “phthalates,” “microplastics,” “heavy metals,” “air pollution,” “PM2.5,” “light pollution,” “mycotoxins” “phytotoxins” “pregnancy,” “maternal,” “fetal programming,” “kidney development,” “nephrogenesis,” “kidney programming,” “DOHaD,” “reprogramming,” “antioxidants,” “melatonin,” “polyphenol,” “vitamin” “hypertension,” and “chronic kidney disease.”

Eligible studies included original research articles, reviews, and clinical studies involving both animal models and human populations that examined the impact of environmental pollutants on kidney disease through oxidative stress mechanisms. Special emphasis was placed on studies addressing redox imbalance, fetal and renal vulnerability, and the therapeutic potential of antioxidants. Reference lists of key articles were also screened to identify additional relevant studies. Figures were generated using Napkin AI software.

## 3. The Interplay Between Oxidative Stress and Kidney Disease

### 3.1. Mechanistic Basis of Oxidative Stress

Oxidative stress occurs when the generation of ROS and RNS surpasses the neutralizing capacity of antioxidant defenses, resulting in redox imbalance [23]. Under physiological conditions, ROS and RNS are generated in controlled amounts as part of normal cellular signaling and play important roles in regulating renal physiology and blood pressure (BP) [24,25]. Key free radicals include the superoxide anion (O_2_•^−^), produced mainly in mitochondria during oxidative phosphorylation, and nitric oxide (NO•), synthesized by nitric oxide synthases (NOS) [26]. In the kidney, major ROS sources include NADPH oxidases (notably NOX4 in tubules), which generate superoxide in renal and vascular cells, and mitochondrial electron transport chain complexes I and III, which leak superoxide during oxidative phosphorylation, especially with dysfunction or pollutant exposure. Xanthine oxidase produces superoxide and hydrogen peroxide during purine metabolism. Additionally, uncoupled NOS generates superoxide instead of NO when cofactors are deficient or when elevated levels of asymmetric dimethylarginine (ADMA), an endogenous NOS inhibitor, promote NOS uncoupling through substrate competition [27]. In inflammation, myeloperoxidase forms hypochlorous acid from hydrogen peroxide, and cytochrome P450 enzymes release ROS during xenobiotic metabolism. Dysregulation of these systems promotes oxidative injury in CKD. While NO• plays an essential role in vasodilation and the regulation of blood pressure and renal hemodynamics [28], its reaction with O_2_•^−^ forms peroxynitrite (ONOO^−^)—a highly reactive RNS that can inflict widespread oxidative and nitrosative damage [29]. Antioxidant defense systems counteract these effects and include enzymatic components (e.g., SOD converting O_2_•^−^ to hydrogen peroxide) and non-enzymatic molecules (e.g., glutathione, vitamins C and E, uric acid) [30].

Excess free radicals cause oxidative modifications to biomolecules: lipid peroxidation disrupts membrane integrity; protein carbonylation alters enzyme activity and receptor function; and oxidative DNA lesions impair transcription and promote mutagenesis. In renal tissues, these molecular insults activate pro-inflammatory pathways (e.g., NF-κB) and pro-fibrotic cascades (e.g., transforming growth factor-β), driving tubular injury, glomerulosclerosis, and progressive nephron loss. Notably, persistent lipid peroxidation can trigger ferroptosis—an iron-dependent, non-apoptotic form of regulated cell death characterized by the accumulation of lethal lipid ROS [31]. Ferroptosis has been increasingly implicated in acute and chronic kidney injury, linking oxidative stress, disorganized iron metabolism, and membrane damage to irreversible nephron loss.

### 3.2. Role of Oxidative Stress in CKD

Oxidative stress plays a central role in the initiation and progression of CKD, functioning both as a pathogenic driver and a potential therapeutic target [32,33]. In CKD, excessive ROS generation arises from multiple sources, including uremic toxin accumulation, chronic inflammation, mitochondrial dysfunction, and activation of the renin–angiotensin system (RAS). This redox imbalance damages lipids, proteins, and nucleic acids, thereby promoting kidney fibrosis, endothelial dysfunction, and progressive nephron loss.

Commonly used oxidative stress markers in CKD encompass indicators of oxidative damage to lipids, proteins, and DNA, as well as measures of antioxidant defense [34,35]. Lipid peroxidation is frequently assessed by malondialdehyde (MDA) [36] or the more specific F2-isoprostanes [37]. Protein oxidation can be evaluated by protein carbonyl content or advanced oxidation protein products (AOPP) [38], while DNA oxidation is typically measured by urinary, serum, or tissue levels of 8-hydroxy-2′-deoxyguanosine (8-OHdG) [39]. These key biomarkers—MDA, F2-isoprostanes, 8-OHdG, and AOPP—are often elevated in CKD and correlate with disease severity and cardiovascular risk.

On the other side, antioxidant status is assessed through total antioxidant capacity (TAC), activities of enzymatic antioxidants such as superoxide dismutase (SOD), catalase (CAT), and glutathione peroxidase (GPx) [40], and levels of non-enzymatic antioxidants, particularly the reduced-to-oxidized glutathione (GSH/GSSG) ratio [41]. Impairments in these antioxidant systems are common in CKD, further exacerbating oxidative injury and contributing to CKD-related morbidity and mortality.

Importantly, oxidative stress is not only a biomarker of kidney injury but also a modifiable therapeutic target. Antioxidant strategies—including dietary natural antioxidants, pharmacologic antioxidants, RAS blockade, and agents targeting mitochondrial ROS—have shown potential in experimental CKD models [42]. However, translating these findings into consistent clinical benefit remains challenging, highlighting the need for biomarker-guided, personalized antioxidant therapies [43]. Advances in non-invasive redox biomarkers and targeted delivery systems could pave the way for precision nephrology approaches aimed at restoring redox homeostasis in CKD.

### 3.3. Oxidative Stress as a Mediator of Kidney Programming

Oxidative stress plays a pivotal role in kidney programming [44,45], the process by which adverse conditions during fetal and early postnatal development shape long-term kidney structure and function, predisposing individuals to CKD later in life [5,6].

A controlled rise in ROS during gestation supports placental blood vessel formation, cellular differentiation, and the development of fetal organs. Conversely, excessive oxidative stress, as seen in complicated pregnancies, is linked to negative maternal and fetal outcomes [46,47]. Accordingly, excessive ROS and RNS can disrupt normal nephrogenesis, leading to low nephron endowment and a spectrum of defects namely congenital anomalies of the kidneys and urinary tracts (CAKUT) [48,49]. Oxidative stress can induce epigenetic modifications [50]—such as DNA methylation, histone modification, and microRNA expression changes—that program gene expression patterns governing nephron number, tubular function, and renal hemodynamics [51]. Transcriptome analysis of ADMA-exposed embryonic kidneys revealed 1221 differentially expressed genes related to kidney development and epigenetic regulation [51]. Likewise, maternal NO inhibition by L-NAME altered 2289 genes in neonatal kidneys [52]. These findings suggest oxidative stress and epigenetic gene regulation during gestation contribute significantly to kidney programming and future kidney disease risk.

Environmental insults such as maternal malnutrition [53], illness [54], inflammation [55], and exposure to pollutants [56] or medications such as glucocorticoids [57] commonly elevate oxidative stress in utero. This increased oxidative burden not only directly impairs nephrogenesis but also promotes hypertension, glomerular hypertrophy, glomerulosclerosis, tubulointerstitial injury, kidney dysfunction, and albuminuria—collectively setting the stage for accelerated CKD progression in adulthood [21].

Kidney programming involves multiple oxidative stress-related mechanisms, including increased ROS-producing enzymes [58], elevated ROS and peroxynitrite levels [58,59], decreased antioxidant capacity [60], higher ADMA concentrations [61], reduced NO bioavailability [61], and enhanced oxidative damage [52]. Biomarkers of lipid peroxidation—such as MDA and F2-isoprostanes—are elevated in offspring kidneys in various rodent models of kidney programming [20,21]. Additionally, 8-OHdG is highly expressed in these models and correlates with adverse renal outcomes. Numerous studies highlight the role of impaired ADMA/NO pathways in oxidative stress–induced kidney programming [62]. Overall, these findings support oxidative stress as a key contributor to kidney programming and CKD later in life, which can be assessed by related biomarkers.

Animal studies reviewed elsewhere [21] show that maternal supplementation with natural antioxidants—including vitamins C and E, amino acids such as arginine and taurine, melatonin, and polyphenols—during pregnancy and lactation can protect kidney development and mitigate the risk of renal programming. In addition to natural compounds, several synthetic antioxidants have also been tested in animal models [63,64,65,66], showing potential to mitigate oxidative stress and preserve kidney function in offspring. Therefore, oxidative stress is both a mediator and amplifier of developmental kidney injury, linking early-life environmental exposures to lifelong kidney health outcomes through the concept of DOHaD. Targeting oxidative stress during these sensitive periods may offer preventive strategies to mitigate programmed kidney disease risk.

## 4. Oxidative Stress Links Environmental Pollutants to Nephrotoxicity

Oxidative stress is a central mediator of kidney injury caused by environmental pollutants. Nephrotoxicity—characterized by increased urinary albumin excretion or reduced GFR—is increasingly linked to chronic exposure to toxicants such as MPs [67], phthalates [68], air pollution [69], and BPA [70]. These agents contribute to kidney dysfunction through oxidative and inflammatory mechanisms beyond traditional risk factors like diabetes and hypertension.

Pollutant exposure leads to cellular uptake via endocytosis, causing intracellular accumulation and excessive ROS production. This disrupts redox balance, triggering lipid peroxidation, mitochondrial dysfunction, DNA damage, and protein modifications, which collectively promote apoptosis and fibrosis [71]. Oxidative stress also weakens antioxidant defenses—including SOD and catalase—and disturbs key signaling pathways such as AMP-activated protein kinase (AMPK) and the phosphoinositide 3-kinase/protein kinase B/mechanistic target of rapamycin (PI3K/AKT/mTOR), aggravating renal tubular epithelial injury [72].

The mitochondrial apoptotic pathway, marked by cytochrome c release and activation of caspase-9 and caspase-3, plays a crucial role in pollutant-induced cell death [73]. As well, disturbances in ion homeostasis and organelle function exacerbate kidney cell damage. Emerging research highlights oxidative stress as a unifying driver of toxicant-associated nephropathy. Given the diverse toxicity profiles of environmental chemicals, each will be discussed in detail below.

### 4.1. Dioxins

Dioxin, most notably 2,3,7,8-tetrachlorodibenzo-p-dioxin (TCDD), is the most toxic and well-studied member of a group of structurally related compounds—including polychlorinated dibenzo-p-dioxins (PCDDs), dioxin-like polychlorinated biphenyls (PCBs), and polychlorinated dibenzofurans (PCDFs) [74]. These persistent organic pollutants are primarily released from anthropogenic sources such as pesticide production, waste incineration, and paper bleaching. Lipophilic in nature, dioxins bioaccumulate in the food chain and persist for years in adipose tissue, enabling chronic, low-level exposure [75]. Experimental and epidemiological evidence indicates that TCDD can induce kidney injury through multiple interrelated mechanisms: activation of the aryl hydrocarbon receptor (AhR) leading to altered gene expression [76], oxidative stress [77], mitochondrial dysfunction, and chronic inflammation; disruption of renal hemodynamics and glomerular filtration; and promotion of tubulointerstitial fibrosis [78].

Maternal exposure to these chemicals—through high-animal-fat diets or occupational contact—may induce epigenetic changes that predispose offspring to long-term CKD [79]. Elevated dioxin exposure has been associated with adverse offspring kidney outcomes in several animal models of kidney programming [80,81,82].

### 4.2. Plastic Chemical Pollutants

Plastics, composed of polymers with additives such as plasticisers, flame retardants, stabilizers, and colorants, are produced in massive volumes, exceeding 400 million metric tonnes annually and projected to triple by 2060 [83,84]. Most additives are not covalently bound and can leach into food, water, and air, entering the body via ingestion, inhalation, or skin contact [85,86]. Many are detected in human biosamples across all life stages [87], yet most of the >16,000 identified chemicals remain unregulated and poorly studied [88]. Major plastic chemical pollutants contribute to kidney disease through a range of toxicological pathways, including oxidative stress, chronic inflammation, endocrine disruption, and direct tubular injury [89,90,91].

#### 4.2.1. Monomers

Major plastic monomers—including bisphenol A (BPA), vinyl chloride, and styrene—are integral to the manufacture of polycarbonate, PVC, and polystyrene, yet also function as endocrine-disrupting chemicals (EDCs) and metabolic toxins. In humans, BPA exposure has been associated with albuminuria and reduced estimated GFR (eGFR) in CKD patients [92], and cross-sectional studies across diverse populations suggest a possible link to hypertension [93]. These renal and vascular effects are thought to be mediated by oxidative stress, podocyte injury, and activation of profibrotic signaling pathways [70]. Mother–child cohort studies further indicate that maternal BPA exposure may be associated with elevated BP in offspring, although findings remain inconclusive [94,95,96]. Vinyl chloride and styrene, both recognized human carcinogens, have been shown in animal models to cause albuminuria, glomerulosclerosis, and tubular injury [97,98,99], yet their kidney-specific effects in humans remain largely uncharacterized.

#### 4.2.2. Plasticizers

Plasticizers, particularly phthalates such as di(2-ethylhexyl) phthalate (DEHP), dibutyl phthalate (DBP), and butyl benzyl phthalate (BBP), are widely used in PVC products and can leach into food, water, and medical devices [100]. As reviewed elsewhere [101], accumulating evidence links phthalate metabolites to proteinuria, decreased kidney function, and higher CKD prevalence, mediated by oxidative stress, mitochondrial damage, altered renal hemodynamics, and activation of inflammatory pathways [102,103,104]. Phthalate metabolites readily cross the placenta, exposing the fetus [105]. Due to their estrogenic and antiandrogenic effects, prenatal phthalate exposure has been associated with adverse renal and cardiovascular outcomes in offspring; epidemiological studies report that elevated maternal urinary DEHP correlates with higher BP, lower estimated GFR, and albuminuria in children [95,106,107]. Regulatory thresholds, as summarized in the 15th Report on Carcinogens (NTP 2021) [108], include an EU tolerable daily intake of 0.05 mg/kg/day, occupational exposure limits of ~5 mg/m^3^, and medical exposures in infants that may reach several mg/kg/day, underscoring pregnancy and early life as the most vulnerable periods. These findings underscore the urgent need to investigate the impact of prenatal phthalate exposure on offspring kidney health.

#### 4.2.3. Flame Retardants and Stabilizers

Flame retardants such as polybrominated diphenyl ethers (PBDEs) and hexabromocyclododecane (HBCD) persist in human tissues for years [109] and are widely used in vehicles, electronics, furnishings, plastics, building materials, polyurethane foams, and textiles [108]. These compounds have been linked to increased oxidative DNA damage in renal cells, disruption of the thyroid–kidney axis, and promotion of tubulointerstitial fibrosis [110,111,112,113]. Stabilizers, including organotins like tributyltin, exert nephrotoxic effects by inducing oxidative stress, apoptosis, and immune dysregulation, characterized by pro-inflammatory cytokine release and immune cell infiltration in the kidney [114]. Ultraviolet (UV) stabilizers such as benzophenones may contribute indirectly to kidney injury through endocrine and metabolic disturbances; in zebrafish models, benzophenones induce oxidative stress-associated renal damage [115,116]. Additionally, nonylphenol and related antioxidant additives display estrogenic activity and provoke oxidative stress in renal tissue [117]. Despite these findings, the impact of these chemicals on kidney developmental programming and the relationship between maternal exposure and offspring renal outcomes remains poorly understood.

#### 4.2.4. Microplastics/Nanoplastics

Plastic degradation produces microplastics (MPs, <0.5 mm) and nanoplastics (NPs, 1–1000 nm) [118]. MPs/NPs can pollute drinking water and accumulate in food chain, which may cause detrimental effects to human body [119]. Collectively, considering both inhalation and ingestion, humans may be exposed to tens of thousands to millions of MPs daily, depending on individual behaviors and environmental factors [120]. Human and animal studies show MPs and NPs can cross the digestive epithelium, translocate to the kidney, and accumulate in kidney tissue [15,121]. Histological analyses confirm renal deposition of particles ranging from 50 nm to 20 μm, raising concerns about potential kidney toxicity.

Critically, MPs serve as carriers for various harmful chemicals and adsorbed environmental pollutants, such as persistent organic pollutants like dioxins and PCBs, facilitating their accumulation in the kidney. Once internalized, they can trigger oxidative stress injury, tubular inflammation, and fibrosis, ultimately contributing to CKD onset and progression [120]. In vitro and in vivo studies show that MPs enter renal cells, triggering mitochondrial ROS overproduction, ER stress, inflammation, apoptosis, and MAPK pathway activation [122]. MP exposure disrupts antioxidant defenses and elevates inflammatory mediators (TNF-α, IL-6, MCP-1), leading to structural damage, lipid accumulation, and fibrosis. Chronic MP exposure can induce ferroptosis—characterized by glutathione depletion, GPX4 suppression, lipid peroxidation, and Fe^2+^ overload—which promotes renal fibrosis [123]. Conversely, antioxidant interventions mitigate oxidative stress and inflammation, highlighting oxidative stress as a central driver of MP-related kidney injury [124,125].

The combined burden of these plastic-associated chemicals—including BPA, phthalates, flame retardants, plastic stabilizers, and MPs/NPs—represents an emerging yet underrecognized risk factor for CKD. The risk is particularly concerning for vulnerable populations such as children, pregnant women, and those with preexisting kidney disease, in whom early-life or cumulative exposures may accelerate CKD onset and progression.

Nanomaterials are not limited to plastics; they encompass metals, metal oxides, carbon-based structures, polymers, and hybrid systems. Nanomaterials are materials with at least one dimension in the nanometer range (1–100 nm) and unique physicochemical properties due to their size. While microplastics and nanoplastics are widely discussed, nanomaterials go far beyond plastics and include a broad variety of inorganic, organic, and hybrid materials. Nanomaterials beyond plastics can cause nephrotoxicity primarily through oxidative stress, inflammation, apoptosis, DNA damage, and structural disruption of renal tissue. The extent depends on physicochemical properties and exposure parameters. While some NPs (like lipid-based carriers) are relatively safe, inorganic and carbon-based NPs pose significant renal risks in preclinical studies.

### 4.3. Heavy Metals

Heavy metals represent a heterogeneous group of inorganic chemical hazards, with lead (Pb), cadmium (Cd), and mercury (Hg) being the most frequently implicated in nephrotoxicity [126,127]. In the general population, lead exposure primarily arises from contaminated air and food, historically linked to leaded petrol emissions and cooking utensils. Cadmium compounds, still used in certain stabilizers and rechargeable nickel–cadmium batteries, contribute to exposure mainly through contaminated household waste, food, and cigarette smoke. Mercury exposure is predominantly dietary, via consumption of contaminated fish, and from dental amalgam. A systematic review of 14 studies reported significant associations between proteinuria and exposure to arsenic, cadmium, lead, and chromium in drinking water [128].

These heavy metals induce oxidative stress through distinct but converging mechanisms that generate ROS/RNS, leading to DNA damage, lipid peroxidation, and protein oxidation [129]. Arsenic binds to critical thiols, produces hydrogen peroxide, and triggers NO–mediated genotoxicity [130]; cadmium depletes glutathione and binds sulfhydryl groups, impairing antioxidant defenses; lead inhibits antioxidant enzymes and disrupts calcium homeostasis, enhancing ROS formation; and chromium undergoes redox cycling, producing superoxide and hydroxyl radicals that form mutagenic lipid peroxidation products [131]. Beyond redox effects, toxic metals impair kidney mitochondria, as shown in 20 studies on arsenic, cadmium, and lead. Metal exposure disrupts mitochondrial membrane potential, electron transport, and bioenergetics, causing oxidative imbalance in a metal- and dose-dependent manner, with the proximal tubule being especially vulnerable [132]. These processes activate redox-sensitive signaling pathways (e.g., NF-κB, AP-1, p53), contributing to nephrotoxicity.

Human studies indicate that lead, cadmium, and mercury tend to accumulate more extensively in the fetal kidney than in the brain [133]. Long-term lead exposure has been associated with the onset of lead-induced nephropathy [134], while cadmium readily enters renal epithelial cells, triggering toxic injury [135]. Mercury, likewise, is capable of inducing acute kidney injury and damaging proximal tubules [136]. In pediatric populations, even sustained low-level exposure to multiple heavy metals has been linked to elevated risks of CKD and hypertension [137,138]. Given that heavy metals remain major environmental and occupational contaminants with well-documented nephrotoxic potential, it is increasingly important to determine whether maternal exposure during pregnancy influences kidney health trajectories into adulthood.

### 4.4. Polycyclic Aromatic Hydrocarbons

Polycyclic aromatic hydrocarbons (PAHs) are lipophilic organic contaminants consisting of two or more fused aromatic rings, generated from industrial processes, vehicular emissions, domestic combustion, and agricultural activities [139]. Their strong fat solubility promotes bioaccumulation in adipose tissue. Among them, benzo[a]pyrene (BaP) is one of the most extensively studied PAHs, well known for its mutagenic, carcinogenic, teratogenic, and immunotoxic effects in humans [140].

PAH-induced nephrotoxicity is driven by oxidative stress from CYP-mediated metabolism to ROS-generating intermediates, causing lipid peroxidation, DNA damage, and mitochondrial dysfunction in renal cells [141]. Activation of the AhR further amplifies pro-oxidant signaling, while depletion of glutathione and inhibition of key antioxidant enzymes weaken renal defense, leading to inflammation and kidney injury [142].

During pregnancy, maternal and cord blood show comparable PAH concentrations, with markedly lower levels in placental tissue, indicating transplacental transfer to the fetus [143]. Postnatally, breastfeeding has been reported to expose 30–95% of infants to PAHs [144]. Gestational PAH exposure has been linked to adverse outcomes including low birth weight, preterm delivery, stillbirth, and congenital malformations [145,146]. In adults, elevated urinary PAH metabolites correlate with reduced eGFR [147], increased BP [148], and albuminuria [149].

### 4.5. Per- and Polyfluoroalkyl Substances

Per- and polyfluoroalkyl substances (PFAS) comprise a broad class of synthetic chemicals widely used in consumer goods and industrial applications [150]. Exposure to PFAS is nearly universal, with compounds such as perfluorooctanoic acid (PFOA) and perfluorooctane sulfonate (PFOS) detected in over 90% of the general population [151]. In adults, elevated serum concentrations of PFOA and PFOS have been correlated with chronic kidney disease (CKD) [152], and similarly, increased PFOA exposure has been linked to impaired renal function in pediatric populations [153]. For pregnant women, ingestion of contaminated food and water, along with airborne exposure, represents the primary routes of PFAS intake. These substances readily cross the placenta and are also transmitted to infants via breastfeeding [151].

Multiple biological pathways have been implicated in PFAS-related renal toxicity, including oxidative stress, disruption of endothelial barrier integrity, and induction of epithelial-to-mesenchymal transition in the kidneys [154]. PFAS exposure leads to increased ROS generation, which damages cellular components including lipids, proteins, and DNA. This oxidative imbalance activates signaling pathways such as peroxisome proliferator-activated receptors (PPARs) and NRF2, affecting cellular metabolism and inflammatory responses [155]. The resulting oxidative stress contributes to endothelial dysfunction, epithelial-to-mesenchymal transition, and tissue injury, especially in the kidneys, linking PFAS exposure to impaired renal function and CKD. Despite growing recognition of PFAS as environmental contributors to kidney dysfunction, the impact of maternal exposure on offspring renal health remains insufficiently understood.

### 4.6. Air Pollution

Air pollution encompasses a mixture of toxic gases and particulate matter in the atmosphere that threaten both human health and environmental quality. These pollutants include a mixture of gases—such as carbon monoxide, nitrogen oxides, sulfur dioxide, ozone—and particulate matter (PM), which consists of tiny solid or liquid particles suspended in the air. PM is categorized by size into coarse (PM10), fine (PM2.5), and ultrafine (PM0.1) particles. Air pollution originates from various sources, including industrial emissions, vehicle exhaust, combustion of fossil fuels, agricultural activities, and natural events like wildfires. Emerging studies have suggested that exposure to air pollution is closely relevant to increased risk of CKD, CKD progression, and ESKD [156,157,158,159]. The mechanism of PM2.5-induced kidney injury involves oxidative stress, inflammatory response, and cytotoxicity [160]. Air pollutant-induced oxidative stress activates redox-sensitive pathways (e.g., AP-1, Nrf2) that initially promote protective antioxidant responses but, with sustained exposure, lead to inflammation and cell death. Vulnerable populations, including the young, elderly, and those with pre-existing conditions, are especially susceptible due to impaired antioxidant capacity, making oxidative stress a central mediator linking air pollution to renal injury.

While the link between maternal exposure to air pollution and congenital anomalies has been documented [161], the impact of early-life exposure to PM on subsequent renal health in offspring remains inadequately understood.

### 4.7. Light Pollution

Unlike chemical or particulate exposures, light pollution primarily disrupts circadian rhythms [162]. Although artificial light is essential for visual performance and safety, growing concerns highlight its potential health and environmental consequences [163]. Chronic exposure to excessive light at night disturbs the sleep–wake cycle, hormonal rhythms (e.g., melatonin, cortisol), and metabolic regulation [162,163,164]. These systemic disturbances can indirectly impair kidney function, tubular reabsorption, and BP control. In addition, circadian disruption promotes oxidative stress and inflammation, directly damaging renal cells and accelerating dysfunction [165,166]. Light-induced circadian misalignment also worsens metabolic and cardiovascular risk factors—such as hypertension, obesity, and insulin resistance—that further exacerbate kidney injury [162,163]. Collectively, these mechanisms suggest that chronic light pollution contributes to kidney disease development and progression through circadian dysregulation and oxidative stress. Importantly, light also regulates circadian signaling pathways critical for pregnancy and fetal development [167,168]. Animal studies indicate that maternal chronodisruption has long-term adverse effects on offspring health, including increased risks of hypertension and kidney disease [169,170,171].

### 4.8. Natural Pollutants

In addition to anthropogenic pollutants, natural toxins such as mycotoxins and phytotoxins can adversely impact kidney health [172]. Mycotoxins, including ochratoxin A and citrinin from contaminated grains and nuts, primarily target the proximal tubules, leading to tubular injury, interstitial fibrosis, and progressive CKD through oxidative stress, mitochondrial dysfunction, and apoptotic pathways [173,174]. Phytotoxins, notably aristolochic acids from certain herbal remedies, induce aristolochic acid nephropathy (AAN), a well-defined nephropathy characterized by tubular atrophy and interstitial fibrosis via mutagenic DNA adduct formation, sustained oxidative stress, and epithelial apoptosis [175,176]. Collectively, these natural pollutants share mechanisms of oxidative stress, mitochondrial damage, and inflammatory activation, emphasizing their role in kidney injury. Although mycotoxin contamination of breast milk has been reported worldwide, data on the effects of lactational transfer on offspring health remain limited [177]. Maternal exposure to aristolochic acids also poses significant risks to fetal and maternal health [178,179], although the direct impact on offspring kidney function remains unclear. Importantly, both mycotoxins and phytotoxins converge on common nephrotoxic mechanisms, including reactive oxygen species generation, mitochondrial injury, impaired DNA repair, and activation of pro-inflammatory and profibrotic signaling cascades, which collectively drive CKD development.

AAN has emerged as a globally recognized form of environmentally induced CKD and urothelial malignancy. First described in the context of “Chinese herb nephropathy,” AAN results from exposure to herbal remedies containing *Aristolochia* species, and is now considered a distinct clinicopathologic entity [180]. Epidemiological studies have linked aristolochic acid exposure not only to cases in East Asia but also to Balkan endemic nephropathy, further highlighting its global importance [181]. The pathophysiology of AAN is driven by the formation of aristolochic acid-DNA adducts that cause characteristic A:T → T:A transversion mutations in tumor suppressor genes, together with persistent oxidative stress, tubular epithelial apoptosis, and inflammatory activation [182,183]. Clinically, AAN presents with an insidious decline in renal function, often progressing rapidly to ESKD. Of note, even brief or low-level exposure to aristolochic acids may cause irreversible nephropathy, emphasizing the urgency for stricter regulation of herbal supplements, food safety monitoring, and environmental surveillance. Recognition of AAN as a model of toxin-induced kidney fibrosis provides important mechanistic insight into how natural pollutants initiate CKD progression.

### 4.9. Other Contaminants

**Figure 1:**
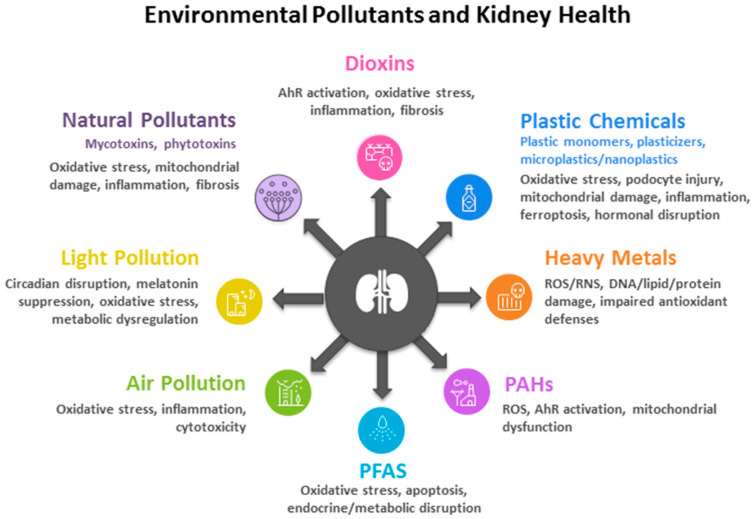
Schematic illustrating mechanisms through which environmental pollutants impair kidney health. Figure created using Napkin AI Image Generator [https://www.napkin.ai/ (accessed on 31 August 2025)].

Emerging contaminants such as nanomaterials extend beyond plastics to include metals, metal oxides, carbon-based structures, polymers, and hybrid systems, each with at least one dimension in the nanometer range (1–100 nm) and unique physicochemical properties [184]. These materials can induce nephrotoxicity through oxidative stress, inflammation, apoptosis, DNA damage, and structural disruption of renal tissue, with the severity depending on their physicochemical characteristics and exposure conditions [185]. Figure 1 summarizes the impacts of environmental pollutants on kidney health.

## 5. Oxidative Stress in Kidney Programming Models of Early-Life Toxicant Exposure

Epidemiological evidence indicates an association between early-life exposure to environmental toxicants and the risk of kidney disease in offspring. However, detailed insights into how such exposures shape kidney programming and contribute to CKD in later life are still limited. Observational data alone cannot establish a direct causal link between prenatal toxicant exposure and adult kidney disease. In addition, these studies provide little understanding of the molecular pathways involved or guidance on potential interventions to counteract adverse programming.

Animal models provide essential platforms for dissecting how oxidative stress contributes to kidney programming following early-life toxicant exposure. These experimental systems not only clarify underlying biological mechanisms but also offer opportunities to explore preventive interventions. Table 1 summarizes representative studies linking toxicant exposure during critical developmental windows with an elevated risk of CKD in offspring [80,121,122,123,124,125,126,127,128,129,130,131,132,133,134,135,136,137,138,139,140,141,142,143,144,145,146,147,148,149,150,151,152,153,154,155,156,157,158,159,160,161,162,163,164,165,166,167,168,169,170,171,172,173,174,175,176,177,178,179,180,181,182,183,184,185,186].

Table 1 shows that rodents are the primary species used in these studies, whereas larger animals have not yet been applied for comparable toxicant exposure experiments. Documented programming effects of environmental toxicants in rats span ages 4 to 21 weeks, roughly equivalent to human childhood through early adulthood [202].

### 5.1. Mechanisms Behind Early-Life Toxicant Exposure Induced Kidney Programming

Early-life exposure to environmental toxicants—including dioxins [80,186,187,188,189], plastic chemical pollutants [190,191,192,193,194], polystyrene nanoplastics [195], heavy metals [196,197], PAHs such as BaP [198], PFOS [199], and air pollution [200,201]—programs kidney development, leading to offspring outcomes characteristic of CKD. These effects include hypertension [186,187,188,198,199,200,201], kidney malformations [189], impaired renal function [190,192,194,195,197], renal hypertrophy [195], and kidney injury [196].

Several mechanisms have been implicated in environmental toxicant–induced kidney programming [5,6]. In addition to oxidative stress, key pathways include aberrant activation of the RAS, reduced nephron endowment, and dysregulation of the AhR signaling pathway, as discussed in the following sections.

### 5.2. Oxidative Stress

Multiple animal models have demonstrated that oxidative stress mediates kidney programming, contributing to hypertension and renal dysfunction in adult offspring [20,21]. Studies have specifically assessed the role of oxidative stress in response to prenatal exposure to toxicants such as TCDD [186,188], BPA [191], and PM2.5 [201]. Markers of oxidative DNA damage, including renal 8-OHdG, were elevated in offspring exposed in utero to TCDD [186] or BPA [191]. In a prenatal PM2.5 exposure model, offspring developed hypertension associated with oxidative stress, which was prevented by the antioxidant tempol [201]. These findings indicate that oxidative stress is a key mechanism linking early-life toxicant exposures to programmed kidney disease and hypertension. Mechanistically, oxidative stress serves as a central hub by increasing ROS through AhR activation, NADPH oxidase activity, mitochondrial dysfunction, and impaired antioxidant defenses, while interacting with pathways such as RAS activation, renal dopamine receptor signaling, and immune modulation. This oxidative milieu contributes to structural kidney alterations, nephron loss, fibrosis, and sustained elevation of BP [20,21,51].

### 5.3. Aberrant Activation of the RAS

The kidney is a primary target of the RAS, which is essential for regulating renal function and BP [203]. Pharmacologic inhibition of RAS remains a cornerstone for managing hypertension and CKD [204]. During nephrogenesis, RAS components are highly expressed, guiding proper renal development and physiological maturation [205]. Disruptions during this critical period—such as maternal insults—can lead to persistent RAS dysregulation, contributing to adult kidney disease and hypertension [206]. Notably, fetal exposure to RAS blockers—including ACE inhibitors, ARBs, and direct renin inhibitors—during the second and third trimesters can induce renal malformations, a phenomenon termed RAS blocker fetopathy [206].

Aberrant RAS activation has been implicated in kidney programming across multiple animal models [207,208]. Several environmental toxicants, including TCDD [188], DEHP [192], and BaP [198], appear to simultaneously affect renal development and RAS activity, resulting in programmed hypertension in adult offspring. Early-life interventions targeting the RAS have shown promise in preventing these adverse outcomes in experimental settings [208]. However, the extent to which oxidative stress mediates interactions between the RAS and environmental chemicals in the developmental origins of kidney disease and hypertension remains to be fully elucidated.

### 5.4. Reduced Nephron Number

Nephron endowment is a fundamental determinant of renal health across the lifespan [209]. Human kidneys typically have around 1 million nephrons, but individual counts can differ by as much as tenfold [210]. Insufficient nephron number, commonly observed in those born with low birth weight (LBW) or preterm, is strongly associated with heightened susceptibility to hypertension and CKD in later life [211,212]. Additional determinants of nephron number include age, sex, and body size. Epidemiologic studies further implicate maternal exposure to environmental toxicants—including plastics, PFOA, PAHs, and fine particulate matter (PM2.5/PM10)—in preterm birth and LBW [213,214,215,216,217], both serve as risk factors for nephron deficit. How such exposures influence nephron endowment and contribute to the developmental origins of kidney disease and hypertension remains an important unanswered question.

Within the framework of the “multi-hit” model of CKD [218], reduced nephron number can serve as an early hit, initiating compensatory hyperfiltration, elevated intraglomerular pressure, and progressive nephron attrition, thereby sensitizing the kidney to subsequent insults. Experimental evidence supports this paradigm: in a maternal DEHP exposure model, offspring developed hypertension and kidney dysfunction alongside disrupted expression of nephrogenesis-related genes [192]. These findings indicate that maternal DEHP exposure interferes with nephron formation, leading to a lasting nephron deficit and adult-onset disease. Although some heavy metals have been implicated in abnormal nephrogenesis [48], their specific role in determining nephron number is not yet established. Future studies should address whether chemical exposures affect nephron endowment in a dose- and developmental stage–dependent manner.

### 5.5. Dysregulated AhR Signaling Pathway

The AhR is a ligand-activated transcription factor that functions as a sensor for environmental chemicals, endogenous metabolites (such as tryptophan derivatives), and certain dietary compounds [219,220]. Once activated, AhR migrates to the nucleus, forms a complex with the aryl hydrocarbon receptor nuclear translocator (ARNT), and regulates downstream genes, including members of the cytochrome P450 enzyme family (CYP1A1, CYP1A2, CYP1B1) and the AhR repressor (AHRR). Through these actions, AhR modulates xenobiotic metabolism, oxidative balance, and immune responses. Aberrant AhR signaling, especially due to maternal exposure to exogenous ligands, has been linked to developmental programming of disease, including kidney disorders [208].

Historically, the first ligands identified for AhR were environmental pollutants such as dioxins, BPA, phthalates, PFOS, and PAHs [219]. Maternal exposure to TCDD, a dioxin, has been shown to induce hypertension in offspring through activation of the AhR/CYP1A1 axis and promotion of TH17-mediated renal inflammation [186]. BPA is likewise an AhR agonist [221], with prenatal exposure leading to offspring hypertension accompanied by increased renal expression of AhR, AHRR, CYP1A1, and ARNT [191].

Phthalates, including DEHP and DBP, also engage AhR signaling and have been associated with impaired renal outcomes in progeny, such as altered kidney function, hypertrophy, and fibrosis [192,193,194]. Gestational PFOS exposure results in offspring hypertension in both sexes by 16 weeks of age [203], though the mechanistic contribution of AhR remains to be fully defined [222]. Similarly, prenatal exposure to fine particulate matter (PM2.5) predisposes offspring to adult-onset hypertension [200,201], with emerging evidence suggesting that AhR mediates its oxidative and inflammatory effects [223].

### 5.6. Epigenetic Dysregulation

Pollutant-induced nephrotoxicity is increasingly recognized to involve aberrant DNA methylation, altered histone acetylation/methylation, and dysregulated non-coding RNAs. Emerging evidence suggests that these epigenetic mechanisms are central to the transgenerational inheritance of pollutant-induced kidney injury. Unlike genetic mutations, epigenetic modifications are reversible and responsive to environmental cues, making them plausible mediators of how toxicant exposures in one generation influence kidney health in unexposed descendants.

In animal models, maternal exposure to TCDD has been shown to induce persistent DNA methylation changes at specific differentially methylated regions (DMRs) within germ cells. When pregnant F0 females were exposed to TCDD, both the F1 and F3 generations were subsequently examined. Importantly, the F3 generation—without any direct exposure—showed an increased incidence of kidney disease in males. Approximately 50 sperm DMRs were identified and associated with this transgenerational kidney phenotype [224].

Emerging evidence demonstrates that Cd exerts its nephrotoxic effects in part through epigenetic modifications. Research reveals that Cd exposure enhances DNA methyltransferase (DNMT1/3a) activity leading to aberrant DNA methylation. Concurrently, Cd promotes histone modifications, including increased H3K9 mono/di-methylation via G9a and altered acetylation states through suppression of the histone deacetylase SIRT1, thereby driving maladaptive gene expression and oxidative stress in renal tubular cells [225]. Beyond DNA methylation, histone modifications play a pivotal role. Cd-induced recruitment of the chromatin reader BRD4, together with enhanced H4K16 acetylation, impairs autophagy and promotes tubular injury. Pharmacological inhibition of BRD4 (e.g., with JQ1) or knockdown approaches restored autophagy and attenuated oxidative injury in Cd-exposed mice [226]. Likewise, inhibition of G9a partially reversed Cd-induced histone methylation and improved cell survival, suggesting the reversibility of these changes [225].

Importantly, epigenetic modifications are not static. Antioxidants such as N-acetylcysteine and resveratrol (a SIRT1 activator) have been proposed to counteract Cd-induced oxidative stress and epigenetic dysregulation [227,228], while their effects in environmental toxicant-induced nephrotoxicity models remain explored. Collectively, these findings underscore the potential of epigenetic reprogramming strategies as novel and reversible approaches to mitigate pollutant-induced nephrotoxicity and reduce long-term kidney risk.

### 5.7. Others

Other molecular mechanisms contributing to kidney programming have been demonstrated in various animal models of developmental origins. These include gut microbiota dysbiosis and perturbations in nutrient-sensing signaling pathways [20,21]. Importantly, these mechanisms do not act in isolation. Many are influenced, directly or indirectly, by environmental toxicants, which can alter microbial composition, interfere with metabolic signaling, and induce heritable changes in gene expression through epigenetic reprogramming [229,230,231].

Such findings suggest that kidney disease and hypertension of developmental origins arise from complex interactions among these mechanisms. While much of this interplay remains to be fully clarified, accumulating evidence underscores their relevance as therapeutic targets. In this context, antioxidant interventions that attenuate oxidative stress may serve as strategies to prevent or mitigate environmental intoxicant exposure-induced kidney programming.

## 6. Antioxidant-Based Strategies to Preserve Kidney Health

The role of antioxidants in human CKD remains uncertain. CKD is a major risk factor for cardiovascular disease and mortality, with oxidative stress as a key contributor. A Cochrane review of 95 RCTs (10,468 patients) found that antioxidant therapy had little or no effect on all-cause or cardiovascular mortality, kidney transplant outcomes, or proteinuria, though it may modestly reduce cardiovascular events, slow CKD progression, and improve kidney function [232]. Study quality was generally low to moderate, results were heterogeneous, and antioxidant use may increase infection and heart failure risk, limiting confidence in their benefit.

Perinatal oxidative stress plays a pivotal role in environmental toxicant-induced kidney programming, leading to adult-onset kidney disease. Excess ROS or RNS may be mitigated by early-life antioxidant interventions, as suggested by preclinical animal models, even though most human trials have not confirmed protection against pollution-induced oxidative stress [233]. Antioxidants can be endogenous—enzymatic or non-enzymatic—or exogenous, including vitamins, minerals, carotenoids, flavonoids, and other dietary compounds [234], and may be classified as natural or synthetic [235].

Animal studies indicate that natural antioxidants—such as vitamins, amino acids, melatonin, and polyphenols—administered during pregnancy and lactation can protect kidney health and prevent kidney programming [20,21]. Plant-based sources, including fruits, vegetables, seeds, and nuts, are rich in vitamins, carotenoids, polyphenols, and glutathione. Synthetic antioxidants have also been tested in animal models. Nutritional programming emerges as a critical mechanism in oxidative-stress-mediated kidney programming, and certain dietary antioxidants have shown efficacy in preventing environmental toxicant-induced adult-onset kidney disease in preclinical studies, as discussed below.

### 6.1. Vitamins and Minerals

Although vitamins are widely recognized as dietary antioxidants, their role in preventing kidney programming induced by environmental toxicants remains uncertain. Vitamins A, C, and E, together with selenium and folate, can protect against oxidative-stress-related kidney injury [236]. Vitamin C acts as a free radical scavenger, whereas vitamin E inhibits ROS-generating enzymes [237,238]. In animal studies, perinatal supplementation with these vitamins—alone or combined with selenium and folate—reduced offspring hypertension triggered by maternal stress or inflammation, key features of kidney programming [239,240,241]. However, excessive intake of certain vitamins, particularly vitamin E at high doses (>400 IU/day) [242], as well as vitamins A and β-carotene, has been associated with increased all-cause mortality. The proposed mechanisms include pro-oxidant effects at supraphysiologic levels, interference with anticoagulation, and disruption of redox-sensitive signaling pathways [243]. Additionally, high vitamin A intake during pregnancy (more than 10,000 units per day or 25,000 units per week) is linked to birth defects [244]. Their potential as reprogramming interventions for toxicant-induced kidney programming has not been tested, and contamination of supplements with heavy metals or other toxic elements poses additional risks [245], underscoring the need for cautious perinatal use, especially under environmental pollutant exposure.

### 6.2. Amino Acids

Several amino acids, including cysteine, taurine, and arginine, possess antioxidant properties [246], and dietary supplementation has been shown to exert therapeutic and protective effects in kidney disease [247,248]. Amino acids play dual roles in environmental nephrotoxicity across the life course. On the one hand, they act as protective modulators: cysteine supports glutathione synthesis, taurine stabilizes membranes and mitigates oxidative stress, and arginine/citrulline sustain NO–mediated vascular and kidney function [249,250,251]. On the other hand, environmental toxicants can disrupt amino acid metabolism—for example, heavy metals deplete sulfur-containing amino acids, while pollutants alter the tryptophan–kynurenine pathway—thereby amplifying oxidative stress, inflammation, and fibrosis [252,253].

During pregnancy, toxicant-induced disturbances in maternal amino acid metabolism or placental amino acid transport can impair nephrogenesis, leading to reduced nephron endowment and greater lifelong CKD susceptibility in offspring [254,255,256]. Notably, maternal citrulline supplementation has been shown to improve NO bioavailability and protect adult rat offspring against kidney programming in a maternal adenine-induced CKD model [257]. Similarly, tryptophan and cysteine have been investigated as reprogramming interventions to prevent offspring hypertension in maternal CKD-related models [258,259]. While these antioxidant amino acids show promise in mitigating kidney programming, their specific effects in toxicant-induced models remain unclear. Given that AhR is a key target of environmental toxicants [219,220] and that tryptophan catabolites act as AhR ligands in the developmental programming of kidney disease [253], tryptophan metabolites hold potential to counteract environmental toxicant–induced kidney injury in offspring, highlighting the need for further studies to define their protective mechanisms.

### 6.3. Polyphenols

Polyphenols represent the most abundant class of bioactive phytochemicals and are widely recognized for their antioxidant and health-promoting properties [260,261]. Among them, resveratrol has been extensively studied; it can chelate transition metals, neutralize free radicals, activate NOS, and enhance the activity of antioxidant enzymes [262]. Because of these actions, polyphenols have attracted attention as potential interventions to support kidney health [263,264].

In the setting of environmental exposure, numerous polyphenols demonstrate nephroprotective actions in experimental models [263,264,265]. Broadly, polyphenols are divided into two categories: flavonoids and non-flavonoids [260]. Flavonoid compounds, such as quercetin, when administered during pregnancy, have been shown to protect offspring from high-fat maternal diet–induced kidney programming and subsequent hypertension [266]. Likewise, epigallocatechin gallate provided during gestation and lactation mitigated the hypertensive effects of prenatal dexamethasone exposure in rat offspring [267].

Resveratrol, a non-flavonoid polyphenol, exerts multiple antioxidant effects, including reducing levels of ROS/RNS, elevating glutathione, upregulating NOS expression, and boosting enzymatic antioxidant defenses [268,269,270]. Beyond its antioxidant profile, resveratrol also functions as an AhR antagonist [271]. In toxicant-induced models of kidney programming—such as those involving TCDD [80,188], BPA [191], and DEHP [193]—maternal resveratrol supplementation improved kidney outcomes in adult offspring. In BPA-exposed pregnancies, for example, resveratrol alleviated offspring hypertension by restoring NO bioavailability, reducing oxidative stress, and suppressing AhR signaling [191]. Emerging evidence suggests that AhR-induced oxidative stress is a key contributor to kidney disease [271]. TCDD-induced hypertension is associated with AhR activation, and resveratrol supplementation during gestation and lactation can mitigate TCDD-induced AhR signaling and oxidative stress. Similarly, perinatal resveratrol therapy reverses maternal BPA exposure–induced increases in AHR protein levels and mRNA expression of AHRR, CYP1A1, and ARNT [191]. Collectively, evidence from animal studies highlights resveratrol and other polyphenols as promising reprogramming agents against environmentally induced kidney disease and hypertension [272].

### 6.4. Melatonin

Melatonin, a tryptophan-derived circadian hormone, supports pregnancy and fetal development while providing antioxidant protection through ROS/RNS scavenging, activation of antioxidant enzymes, and restoration of NO signaling [273,274,275,276]. Light pollution suppresses nocturnal melatonin secretion, resulting in circadian misalignment, oxidative stress, and metabolic dysregulation [162,163,164]. During gestation, reduced maternal melatonin may impair fetal programming, decrease nephron endowment, and increase susceptibility to kidney disease in later life. Evidence from animal studies indicates that perinatal melatonin supplementation can prevent kidney programming across diverse experimental models [168,275]. Although melatonin is generally safe in children, its use during pregnancy is not currently recommended [277,278]. Further research is needed to determine whether restoring melatonin signaling can counteract kidney programming induced by light pollution as well as other environmental toxicants.

### 6.5. Synthetic Antioxidants

**Figure 2:**
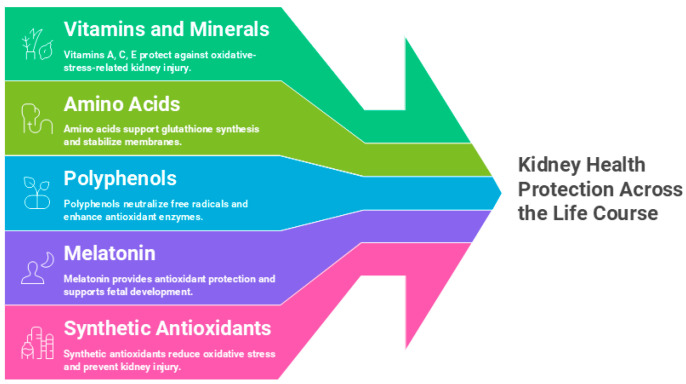
Antioxidant-based approaches for preserving kidney health against environmental pollutants across the life course. Figure created using Napkin AI Image Generator [https://www.napkin.ai/ (accessed on 31 August 2025)].

Alongside natural antioxidants, several synthetic compounds have been studied in kidney disease [232]. MitoQ, a coenzyme Q10 analog, reduces oxidative stress by suppressing superoxide and lipid peroxidation, and perinatal treatment prevented hypertension and kidney injury in offspring of smoking-exposed mice [279]. Dimethyl fumarate, an Nrf2 activator, improved renal outcomes in a dexamethasone–high-fat diet model by lowering 8-OHdG while enhancing NO [280]. Tempol, a SOD mimetic, also show protective effects in experimental hypertension [281]. NAC, a synthetic L-cysteine analog and glutathione precursor, has demonstrated perinatal protection against kidney programming in various animal models [21,282], but its role in toxicant-induced kidney programming remains untested. To date, none of these synthetic antioxidants have been applied in clinical practice during pregnancy. Figure 2 illustrates potential antioxidant-based strategies to counter kidney programming and preserve kidney health in the face of environmental pollutants.

## 7. Conclusions and Future Perspectives

**Figure 3:**
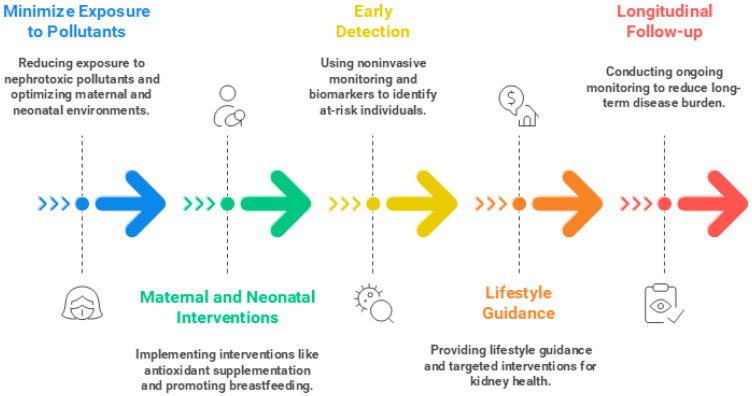
A life-course approach to the prevention of environmental nephrotoxicity. Figure created using Napkin AI Image Generator [https://www.napkin.ai/ (accessed on 31 August 2025)].

Preventing environmental nephrotoxicity requires a life-course-oriented strategy that integrates risk reduction, early intervention, and ongoing monitoring [283,284] (Figure 3). At the earliest stage, minimizing exposure to nephrotoxic pollutants and optimizing maternal and neonatal environments—including limiting light pollution to preserve circadian rhythms—represents a critical first line of defense. Interventions during pregnancy and early life, such as maternal antioxidant supplementation (e.g., vitamins C and E) [285,286] or promotion of exclusive breastfeeding [287,288,289], may help counteract oxidative-stress-driven kidney programming. Early detection through noninvasive monitoring and the use of oxidative stress biomarkers can identify fetuses, neonates, and children at risk, enabling timely preventive or therapeutic measures [290]. Later in life, lifestyle guidance, targeted interventions, and longitudinal follow-up remain essential to protect kidney health and reduce long-term disease burden [291].

Despite promising preclinical evidence, translation into human practice remains limited. Most data on antioxidant use to prevent pollutant-induced kidney damage in pregnancy are derived from animal studies, providing mechanistic insights but limited direct applicability. While antioxidants can reduce oxidative stress in CKD patients [292,293], none are currently recommended in clinical guidelines, and careful attention to compound, dose, and timing is needed to avoid pro-oxidant effects—for example, vitamin E may increase oxidative stress if co-antioxidants like vitamin C are insufficient [294]. Candidate biomarkers (e.g., urinary 8-OHdG, F2-isoprostanes, glutathione redox ratio) may help identify individuals with elevated oxidative burden who are most likely to benefit from targeted antioxidant therapy. These limitations highlight the need for well-designed human studies to guide safe antioxidant interventions during pregnancy and early life.

Early-life oxidative stress remains a major driver of kidney programming, yet maternal and neonatal interventions have been underexplored, and human studies are sparse, particularly in fetuses and neonates. Breastfeeding, rich in natural antioxidants, represents a safe and potentially effective preventive measure that warrants further study [287,288,289].

The development and validation of reliable oxidative stress biomarkers is crucial to guide intervention. Although no single biomarker is ideal, panels that capture the pathogenic processes observed in animal studies may improve early detection of kidney injury [295,296]. Non-coding RNAs, which are increasingly recognized as key regulators of kidney disease and epigenetic modifications [297], also represent innovative candidates for biomarkers in environmental nephrotoxicity and CKD. Emerging technologies, such as liquid biopsies, may further facilitate rapid and noninvasive monitoring of oxidative-stress–related kidney damage [298].

Overall, optimizing kidney outcomes requires a comprehensive, perinatal-focused approach: reducing environmental exposures, applying evidence-based antioxidant strategies, and integrating validated biomarkers for early risk identification. This integrated framework bridges preclinical findings with clinical practice, offering a pathway to prevent environmentally induced kidney disease across the life course.

## Figures and Tables

**Table 1:** Summary of Animal Models Linking Early-Life Toxicant Exposure and Kidney Programming.

Toxicant	Exposure Period and Dose	Species	Age at Evaluation (Weeks)	Kidney Programming	Ref
TCDD	200 ng/kg orally at GD14/21 and PND7/14	SD rats/M	12	Hypertension	[186]
TCDD	200 ng/kg, 4 oral doses across gestation and lactation	SD rats/M	12	Hypertension	[80,187]
TCDD	200 ng/kg, 4 oral doses across gestation and lactation	SD rats/M	16	Hypertension	[188]
TCDD	6 µg/g orally at GD 14.5	C57BL/6N mice/M	12	Kidney malformation	[189]
BPA	10/100 mg/kg/day during GD9–16	OF1 mice/M & F	5	Disturbed kidney function	[190]
BPA	50 mg/kg/day across gestation and Lactation	SD rats/M	16	Hypertension	[191]
DEHP	0.25/6.25 mg/kg/day during pregnancy	Wistar rats/ M & F	21	Disturbed kidney function and renal hypertrophy	[192]
DEHP	10 mg/kg/day across pregnancy and lactation	SD rats/M	12	Hypertension	[193]
DBP	850 mg/kg/day during GD14–18	SD rats/M	8	Disturbed kidney function and renal fibrosis	[194]
NPs	1 mg/L polystyrene-NPs in water across pregnancy and lactation	C57BL/6 J mice/M & F	4	Disturbed kidney function and renal hypertrophy	[195]
Cd	Cd chloride 2.0/2.5 mg/kg/day at GD8, 10, 12 & 14	SD rats/M	7	Kidney injury	[196]
Cd	Cd chloride 0.5 mg/kg/day during pregnancy	Wistar rats/ M & F	8	Disturbed kidney function	[197]
BaP	600/1200 mg/kg/day during GD14-17	LEH rats/M & F	8	Hypertension	[198]
PFOS	50 μg/mL from GD4 to delivery	SD rats/M & F	16	Hypertension	[199]
PM_2.5_	PM2.5, 16 wk prior to delivery	C57BL/6N mice/M & F	12	Hypertension	[200]
PM_2.5_	Oropharyngeal PM2.5, 1.0 mg/kg, GD8, 10, 12	SD rats/M	14	Hypertension	[201]

GD, gestational day; PND, postnatal day; TCDD, 2,3,7,8-tetrachlorodibenzo-p-dioxin; BPA, bisphenol A; DEHP, di-2-ethylhexylphthalate; DBP, di-n-butyl phthalate; PFOS, perfluorooctane sulfonic acid; BaP, benzo(a)pyrene; NPs, nanoparticles; Cd, cadmium; PM_2.5_ (particulate matter < 2.5 mm); SD, Sprague-Dawley rat; OF1, Oncins France 1; LEH, Long Evans Hooded; M, male; F, female.

## Data Availability

Data are contained within the article.
